# Production and Characterization of Active Bacterial Cellulose Films Obtained from the Fermentation of Wine Bagasse and Discarded Potatoes by *Komagateibacter xylinus*

**DOI:** 10.3390/polym14235194

**Published:** 2022-11-29

**Authors:** Patricia Cazón, Gema Puertas, Manuel Vázquez

**Affiliations:** Department of Analytical Chemistry, Faculty of Veterinary, University of Santiago de Compostela, 27002 Lugo, Spain

**Keywords:** bacterial cellulose, agricultural waste, *Komagateibacter xylinus*, active film, antioxidant properties, ABTS, DPPH, TPC

## Abstract

Potato waste, such as peels, broken or spoiled potatoes and grape bagasse residues from the winery industry, can be used for the biotechnological production of high-value products. In this study, green, sustainable and highly productive technology was developed for the production of antioxidant bacterial cellulose (BC). The aim of this work was to evaluate the feasibility of a low-cost culture medium based on wine bagasse and potato waste to synthesize BC. Results show that the production of BC by *Komagateibacter xylinus* in the GP culture medium was five-fold higher than that in the control culture medium, reaching 4.0 g/L BC in 6 days. The compounds of the GP culture medium improved BC production yield. The mechanical, permeability, swelling capacity, antioxidant capacity and optical properties of the BC films from the GP medium were determined. The values obtained for the tensile and puncture properties were 22.77 MPa for tensile strength, 1.65% for elongation at break, 910.46 MPa for Young’s modulus, 159.31 g for burst strength and 0.70 mm for distance to burst. The obtained films showed lower permeability values (3.40 × 10^−12^ g/m·s·Pa) than those of other polysaccharide-based films. The BC samples showed an outstanding antioxidant capacity (0.31–1.32 mg GAE/g dried film for total phenolic content, %DPPH^•^ 57.24–78.00% and %ABTS^•+^ 89.49–86.94%) and excellent UV-barrier capacity with a transmittance range of 0.02–0.38%. Therefore, a new process for the production of BC films with antioxidant properties was successfully developed.

## 1. Introduction

In 2019, the Food and Agriculture Organization of the United Nations estimated that approximately 14% of the world’s food, valued at 400 billion USD, is lost on an annual basis between harvesting and the retail market (https://www.fao.org, (accessed on 24 October 2022)). Food loss and waste has indeed become an issue of great public concern. The 2030 Agenda for Sustainable Development reflects the increased global awareness of the problem. Target 12.3 of the Sustainable Development Goals calls for halving global per capita food waste at retail and consumer levels by 2030, as well as reducing food losses along production and supply chains (https://www.fao.org, (accessed on 24 October 2022)). Food loss and waste streams often contain valuable components that should be considered to transform the current linear supply chain into a circular model, following the foundations of a circular economy. In addition, these resources can help address the major global challenge of transitioning from a fossil-based to a bio-based economy, including energy, chemicals and materials. Food loss and waste have been assessed mainly for biofuel production and energy recovery and, to a lesser extent, for synthesizing bio-based materials. Due to the need to find biodegradable materials to help minimize the consumption of non-biodegradable packaging with a higher environmental impact, there has been an increased interest in evaluating these wastes as feedstock for the production of biomaterials [1]. Often, food loss and waste can be used directly without separation or purification processes as a source of nutrients for the fermentation of microorganisms that produce biopolymers such as polyhydroxyalkanoates or bacterial cellulose (BC), reducing production costs [2,3].

BC, like vegetable cellulose, is a linear polysaccharide of covalently linked glucose residues between carbon 1 and 4 (β1–4). BC has several advantages over vegetable cellulose: (1) BC is synthesized in a pure form, free of other vegetable impurities; (2) it does not require costly extraction and purification processes or the use of environmentally hazardous chemicals; (3) it has a higher degree of crystallinity and polymerization; (4) it has superior tensile properties; and (5) it has a higher specific area and swelling capacity due to its network structure [4]. BC is presented as a promising biodegradable material with applications in multiple fields, such as biomedicine [5], pharmaceuticals, cosmetics and the food industry [6,7], among others. However, the large-scale industrialization and commercialization of BC remains a challenge due to high fermentation costs, low productivity and expensive culture media. In order to reduce the costs of culture media, research has been intensifying to evaluate low-cost biomass waste substrates as a nutrient source for BC production [8]. Among waste generated in greater volumes and due to their chemical compositions, waste from wineries and the potato industry can be excellent candidates for BC production.

The International Organization of Vine and Wine estimated that, in 2018, the global production of grapes reached 77.8 Mt, being 57% of the harvest destined for wine production (https://www.oiv.int, (accessed on 24 October 2022)). During the production of red wine, after fermentation and pressing, a solid residue is obtained consisting of stalks, skins and seeds, namely grape pomace or grape marc. Grape marc represents approximately 20% of the total processed grapes and is rich in polyphenols, anthocyanins, flavonoids, essential oils and polysaccharides, with potential revaluation applications [9]. The rich composition of wine residues in these components allows value-added products to be obtained through biotechnological processes [9]. Previous studies have evaluated the use of grapes in several versions (juice, must and unpressed residue) as a cheap carbohydrate source to produce BC. Mostly unpressed residues or grape extract with a high content of free monosaccharides are used as alternative carbon sources in Hestrin–Schramm media or in alternative media enriched with corn steep liquor as a nitrogen source [10,11,12,13]. However, most waste generated in wineries is grape marc, which, after fermentation and pressing, has a limited content of monosaccharides available for fermentation. On the other hand, in many studies, bagasse components (seeds, stalk, pulp or skin) are separated and subjected to separation processes in order to use mainly the components with the highest monosaccharide content [10,12]. Additional operating units increase the cost of production. In this work, the whole residue is evaluated, avoiding the separation of components, as a source of monosaccharides and nutrients that improve the yield of BC production.

The potato (*Solanum tuberosum*), which is the fourth leading crop after rice, wheat and maize, plays an important role in the human diet worldwide. The worldwide production of potatoes is constantly increasing and currently amounts to around 380 Mt. The share of potatoes in 2011 designated as a raw material for the industry was 130 Mt [14,15]. The main compound of potato is starch. It is composed of two polysaccharides: the linear molecule amylose, which consists of glucose polymers connected predominantly by α-1,4 bonds, and amylopectin, the main macromolecular component of starch and responsible for the structure of starch granules [16]. The contribution of potato starch to total starch production in the EU is 15–20% and 3–4% worldwide. In recent years, potato starch production in Europe has been approximately 1.7 million tons per year [17]. Due to their content of starch, cellulose, hemicelluloses and fermentable sugars, potato residues (non-commercial potatoes, potato peelings, potatoes with high content of glucoalkaloids α-solanine, and α-chaconine) are a good substrate for biotechnological processes [16]. A previous study evaluated the use of potato peel waste [18] for the low-cost production of BC. In that case, the culture medium contains only sugars from the acid hydrolysate of the potato peels, without being enriching with other components or hydrolysates of other residues with a high content of micronutrients or antioxidants that can increase the yield of BC production or that can obtain functionalized films with antioxidant properties.

The acid hydrolysis treatment of potato and wine bagasse residues is a simple and economical option to break down cellulose and hemicellulose structures and to increase the content of fermentable monosaccharides.

The use of the potential high content of hydrolysable polysaccharides to obtain fermentable sugars, as well as the possible presence of micronutrients, mainly in the remains of grape bagasse, could turn these agri-food wastes into an excellent source of substrates to obtain enriched culture media for economically more profitable biotechnological processes, such as the production of BC. Moreover, the presence of antioxidant components in the bagasse could allow in situ modifications of the BC films, retaining these antioxidant components within the BC polymeric matrix, leading to films with antioxidant properties after fermentation, purification and drying processes.

The aim of this work was to evaluate the feasibility of using a low-cost culture medium based on Garnacha bagasse and potato waste to synthesize BC. The transfer of the antioxidants of the bagasse extract to the polymeric matrix was evaluated to obtain active BC films with antioxidant properties. Moreover, the mechanical, permeability, swelling, antioxidant and optical properties of the obtained films were measured.

## 2. Materials and Methods

### 2.1. Chemicals and Standards

For the synthesis of BC, a pure freeze-dried culture of *Komagateibacter xylinus* was obtained from the Spanish Type Culture Collection “Colección Española de Cultivos Tipo” (CECT) (Paterna, Spain). The control medium was prepared with D(+)-glucose monohydrate (99%) provided by Acros Organics (Geel, Belgium) and yeast extract supplied by Scharlau (Barcelona, Spain). The natural culture medium that was evaluated was based on bagasse extracts and potato hydrolysates obtained under acid hydrolysis conditions using sulfuric acid (95–97%), and sodium carbonate purchased from Scharlau Microbiology (Barcelona, Spain) was used to neutralize the media.

Grape pomace from *Vitis vinifera* L. Garnacha Tintorera, which contains skins, pulp, seeds and stems after the pressing process in winemaking, was kindly provided by a local winery (42°33′58.2″ N 7°40′37.4″ W) in the Ribeira Sacra region (Lugo, Spain). Tuber samples of potatoes (*Solanum tuberosum*) were generously supplied by a local company called Pitita’s Farm (Dozón, Spain), harvested at the following geocoordinates: 42°36′14.0″ N 8°01′24.6″ W.

The synthesized BC samples were purified using sodium hydroxide (98%) and were conditioned using anhydrous sodium bromide (99%) or silica gel from Acros Organics (Geel, Belgium). All reagents and solvents used for HPLC were of analytical HPLC grade. Standards of gluconic acid, D(+)-glucose monohydrate (99%), D-xylose (99%), L(+)-arabinose (99%), 5-(hydroxymethyl)furfural (98%) and furfural (99%) standards were purchased from Acros Organics (Geel, Belgium). Folin–Ciocalteu reagent from Panreac (Barcelona, Spain), 1,1-diphenyl-2-picrylhydrazyl radicals (DPPH^•^) and 2,2′-azino-bis(3-ethylbenzothiazoline-6-sulfonic acid) (ABTS^•+^) free radical reagents purchased from Alfa Aesar (Haverhill, MA, USA) and methanol and ethanol provided by Scharlau Microbiology (Barcelona, Spain) were used for antioxidant assays.

### 2.2. Culture Media for Bacterial Cellulose Synthesis

Grape pomace, which contained skins, pulp, seeds and stems, was dried using hot air-drying at 50 °C for 24 h. The dried grape pomace was ground into powder and was sieved by a 0.5 mm mesh sieve. The homogeneous sample with a size lower than 0.5 mm was stored in an airtight container at room temperature until use. The same process was followed to dry and homogenize the potato samples after washing and slicing. Aliquots of the homogenized raw material were analyzed for moisture determination by drying a known amount of sample at 105 °C to a constant weight. The moisture content test was carried out in triplicate.

The sulfuric acid hydrolysis was performed in an autoclave capable of controlling temperature (±1 °C). Samples with a bagasse powder/potato powder ratio of 50:50 (*w*/*w*) were prepared in 0.5 L bottles by adding a 2% sulfuric acid solution to a final solid/liquid ratio of 1:10. In order to allow all the matter to be wetted and to obtain good supernatant recovery, the mixture was kept under vigorous stirring for 1 h before the thermal process. Then, the reaction mixture was submitted at 125 °C for 1 h. When the heat treatment was completed and the mixture was cooled to room temperature, the hydrolysate liquors were neutralized with CaCO_3_ until reaching a pH of 6. The resulting solution was filtered through filter paper (10 µm porosity) to remove un-dissolved material, and the CaSO_4_ was precipitated from the supernatant. The natural grape pomace–potato (GP) culture media were prepared with the hydrolysates and were supplemented with 10 g of yeast extract/L.

The selected synthetic culture media were recommended by the Spanish Type Culture Collection (CECT), equaling the glucose concentration of that obtained in the natural medium studied and with 10 g of yeast extract/L.

### 2.3. Bacterial Cellulose Fermentation

Pre-inoculum was prepared from a starter stock of *K. xylinus*. For this purpose, 100 mL of GP culture medium and 100 mL of control synthetic culture medium were prepared in 250 mL Erlenmeyer flasks and were sterilized at 121 °C for 15 min. The sterile media were inoculated with the *K. xylinus* starter culture and were incubated under static conditions for 2 days at 30 °C.

For the BC production study, Petri dishes were prepared with 25 mL of culture medium (GP or control) and were inoculated with 10% of the volume of the corresponding pre-inoculum. The dishes were incubated under static conditions for a maximum of 10 days at 30 °C. Each day, 3 dishes were removed to analyze the composition of the culture medium and the BC film formed in the air–liquid medium. All experiments were carried out in triplicate, and the means and standard deviations are given. The parameters that were measured to analyze the production of BC in the media under study were as follows:

- The pH was measured using pHmeter pH 210 (Hanna Instruments, Woonsocket, RI, USA).
- The drained weight (g) was determined from the weight of the samples of BC, from which excess liquid was removed by draining the BC for 1 h.
- The dry weight (g) of the BC was determined from samples dried at 105 °C for 24 h.
- The swelling capacity (%S) of the polysaccharides was calculated from the difference in the drained weight and the dry weight of the polymer matrix.
- BC concentration (g/L) was calculated by considering the amount of BC synthesized (dry weight, g) per unit of volume of the culture medium in each Petri dish (25 mL).
- BC productivity (g/L·h), BC yield and BC efficiency were calculated using Equations (1)–(3) [19,20]:

BC productivity (g/L·h) = X/V·t(1)

BC yield = (X − X_0_)/S_0_ − S (2)

BC efficiency = (X − X_0_)/S_0_ (3)

where *X* is the biomass concentration under steady-state conditions (g/L), V is the reaction volume (L), t is the time of reaction (d), X_0_ is the initial biomass concentration (g/L), S is the glucose concentration (g/L) and S_0_ is the initial glucose concentration (g/L).

### 2.4. Analytical Methods

Glucose, gluconic acid, cellobiose, xylose, arabinose, hydroxymethyl furfural and the furfural content of the culture media were determined by HPLC using a Rezex RHM (Phenomenex, Torrance, CA, USA) column with isocratic elution (flow rate of 0.400 mL/min and mobile phase of 0.025 M H_2_SO_4_), a column oven set at 45 °C and a refractive index detector (RI) (LC 2000 plus, Jasco, Tokio, Japan). The RI detector was used for the analysis of glucose, cellobiose, xylose and arabinose. Gluconic acid, furfural and HMF were detected spectrophotometrically with DAD. Gluconic acid was detected at 220 nm, and furfural and HMF were detected at 275 nm [21,22,23]. The analysis was performed in triplicate by taking 3 Petri dishes each day until the end of the incubation (10 days).

### 2.5. Bacterial Cellulose Films Processing

Based on the optimal conditions of the minimum time required to achieve the highest BC production observed in the fermentation study in synthetic and alternative media, *K. xylinus* was fermented under static conditions at 30 °C. After the fermentation time, the formation of a cellulose film floating on the surface of the culture medium was observed. The pellicles were separated from the culture media and were washed with water to remove the culture medium remains. The homogeneous BC films were treated with a solution of NaOH 1% (*w*/*v*) at 90 °C for 1 h to remove bacterial cells. Later, films were subjected to several washing cycles with distilled water until a pH of 7 of the wash water was reached. The wet BC pellicles were placed between a support designed to dry the samples and to prevent them from wrinkling or shrinking. The films were dried for 48 h at room temperature. Finally, the films were cut into a specific size, and the thickness of each sample was measured at 5 random points using a thickness meter ET115S (Etari GmbH, Stuttgart, Germany). The samples were stored in desiccators with a saturated anhydrous sodium bromide solution or silica gel for 5 days, which was enough time to ensure that the samples reached an equilibrium moisture content.

### 2.6. Characterization of the Antioxidant Bacterial Cellulose Samples as Films

The antioxidant capacity of the samples was determined by the ABTS^•+^ (2,2′-azino-bis (3-ethylbenzothiazoline-6-sulfonic acid) and DPPH^•^ (1,1-diphenyl-2-picrylhydrazyl radicals) free radical scavenging assays, and the total phenolic content (TPC) was determined by the Folin–Ciocalteu method, as described elsewhere [24,25]. The TPC was expressed as mg of gallic acid equivalents (GAE) per 1 g of dried BC sample determined by calibration with gallic acid. The obtained calibration equation was TPC (mg ac gallic/g sample) = 1010 Abs_765nm_ − 85.311, and the determination coefficient was R^2^ = 0.9993.

The equilibrium moisture content (%W) and the swelling capacity (%S) were estimated by gravimetric methods, following the previously reported method [26,27]. Water vapor permeability (WVP) was measured following the ASTM Standard Test Method E96 (https://www.astm.org/Standards/E96.htm, (accessed on 20 October 2022)). The WVP of the samples was determined at 30 °C and 50% relative humidity for 4 h, which was enough time to reach a dynamic equilibrium in the water flux. The equilibrium moisture content and WVP tests were carried out in triplicate.

The tensile strength (TS, MPa), percentage of elongation at break (%E, %), Young’s Modulus (YM, MPa), burst strength (BS, g) and distance to burst (DB, mm) were measured with a texturometer (TA-XT plus, Stable Micro System, UK). The tensile test was performed using the standard method D-882 (ASTM), and samples had a size of 15 × 100 and were preconditioned for 5 days at room temperature and 57% relative humidity. The samples were clamped between the grips of the texturometer with an initial gap of 40 mm and a test speed of 1 mm/s. Seven samples were tested to calculate the mean value of each parameter [28,29]. A film holder (HDP/FSR) attached to the texturometer was used for the puncture test. Sample squares with 30 mm sides were conditioned at 57% relative humidity and room temperature for 5 days. The test was carried out in triplicate with a cylindrical probe (d = 3 mm) at a constant crosshead speed of 1 mm/s until rupture.

The UV barrier, transparency, opacity and color of the samples were measured with absorbance values in the UV-Vis light range (190–800 nm) using a spectrophotometer, V-670 (Jasco Inc., Tokyo, Japan), as described elsewhere [29]. The transparency and opacity values of the samples were estimated using their transmittance and absorbance values at 500 nm and 600 nm wavelengths. Spectra Manager software (Jasco Inc., Tokyo, Japan) was used to determine the CIE L*a*b* coordinates.

### 2.7. Statistical Analysis

The obtained results were statistically analyzed by a one-way analysis of variance (ANOVA) by employing Microsoft Excel^®^ software. Differences between pairs of means were assessed based on confidence intervals using the Tukey’s post hoc test. The least significant difference was *p* < 0.05.

## 3. Results and Discussion

The moisture contents of bagasse powder and potato powder dried with the air-dryer were (2.38 ± 0.06)% and (7.72 ± 0.06)%, respectively. These values were considered when preparing the hydrolysis mixtures.

**Figure 1:**
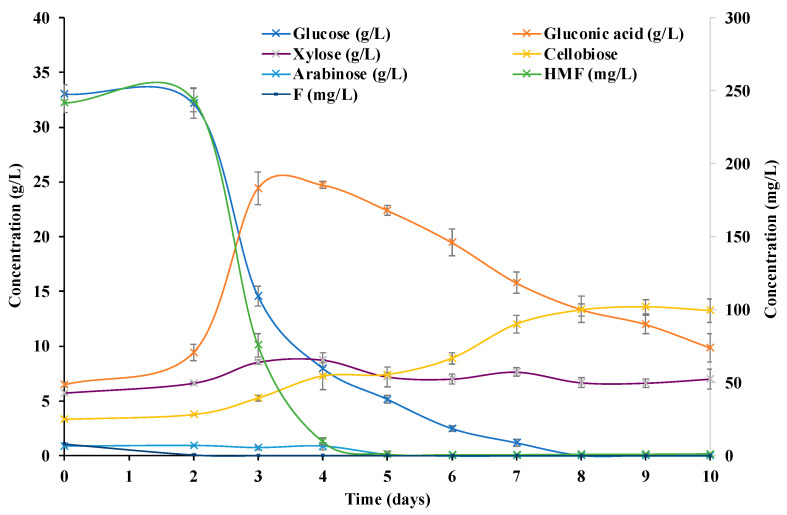
Cellobiose, gluconic acid, glucose, xylose, arabinose, 5-(hydroxymethyl)-2-furaldehyde (HMF) and furfural (F) concentration (g/L; mg/L) of the bagasse–potato culture medium during the incubation time. Values are shown as means and standard deviations.

**Figure 2:**
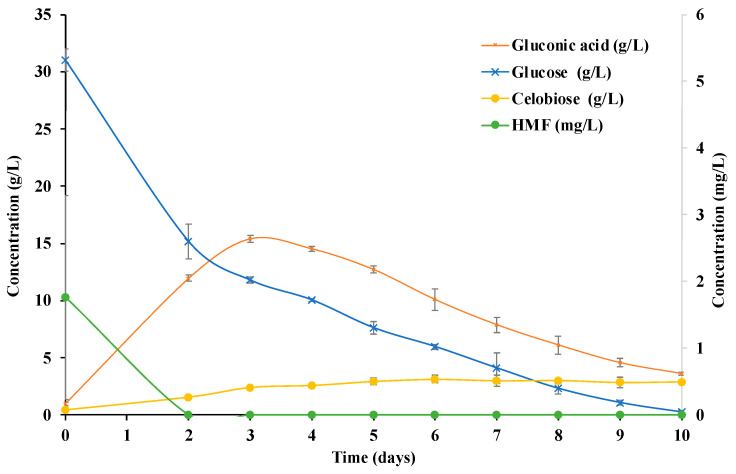
Cellobiose, gluconic acid, glucose and 5-(hydroxymethyl)-2-furaldehyde (HMF) concentration (g/L; mg/L) of the control culture medium during the incubation time. Values are shown as means and standards deviation.

A minimum of 36 Petri dishes were prepared for the tests analyzed with the GP culture medium, and the same number of Petri dishes was prepared for the control culture medium. Every 24 h, from the start time of the incubation of *K. xylinus*, three plates were removed to evaluate and measure the different parameters that determined the changes in the composition of the culture medium and the growth of the BC. Figure 1 and Figure 2 show the composition changes in the main analyzed components of the GP medium and the control culture medium throughout the incubation period. Table 1 and Table 2 show the values obtained for the different parameters related to the production of BC in the GP and the control medium, respectively.

### 3.1. Bacterial Cellulose Production

The production of BC was tested on pure media of Garnacha bagasse hydrolysates. However, no BC production was observed in pure Garnacha bagasse media. The HPLC analysis of the carbon sources of Garnacha hydrolysate revealed a low concentration of sugars such as glucose (<5 g/L), xylose (<5 g/L) or arabinose (<2 g/L). Low concentrations of the carbon sources were likely not enough to form homogeneous BC films on the plate surfaces. The content of monosaccharides in the bagasse was low because the many sugars were previously consumed by yeasts during alcoholic fermentation in the wine production process. The used bagasse was previously drained and pressed, and therefore, almost all the juice with free sugars were extracted, being the final residue of the winemaking process. In other studies, a concentration of monosaccharides up to 17.6% or 13.64% was observed in grape extracts obtained directly from grape juice extract enriched with sugar cane (5%) [13] or from grapes under enzymatic hydrolysis treatment [2]. Furthermore, the content of monosaccharides and total sugars in grapes depends on the type of variety and the part of the grape analyzed [30].

Despite the low content of free monosaccharides, this extract contains a high content of phenolic compounds, antioxidant components or micronutrients as vitamin C that are of great interest to be included in active biodegradable films [2,31,32]. Moreover, these components can enrich the culture medium and enhance the synthesis of polysaccharides, such as BC [2,33]. The evaluated Garnacha hydrolysate showed a TPC value of 79.3 ± 6.7 mg GAE/L of extract, the %DPPH^•^ was (37.4 ± 6.9)% and the %ABTS^•+^ was (30.0 ± 6.2)%, indicating valuable content of phenolic compounds and other antioxidants in the bagasse hydrolysates.

The enrichment of Garnacha bagasse hydrolysate with non-commercial potato waste hydrolysate may increase the monosaccharide content (mainly glucose) of the culture medium to enhance the growth of *K. xylinus* and the BC production. A previous work evaluated the acid hydrolysis with sulfuric acid of non-commercial potatoes to obtain glucose for fermentative purposes [16]. The data showed a glucose concentration of 73.08 ± 0.09 g/L after a hydrolysis treatment at 130 °C for 60 min with a 2% sulfuric acid solution and a solid/liquid ratio of 1:10 [16].

Based on these results and with the goal of improving the monosaccharide content of the culture medium, culture media enriched with potato residues were prepared with a bagasse:potato ratio of 50:50 (*w*/*w*). The selected hydrolysis conditions were like the previous work, at 125 °C for 60 min with a sulfuric acid concentration of 2%. A slightly lower hydrolysis temperature was chosen to minimize the formation of undesirable components in the hydrolysate, such as HMF or furfural.

Figure 1 and Figure 2 show the content of the main monosaccharides and disaccharides in the fermentation of *K. xylinus* in the GP and the control culture media. Starting from a GP hydrolysate of 50:50 (*w*/*w*), a medium with a glucose concentration of 33.0 g/L, an arabinose concentration of 0.9 g/L and a xylose concentration of 5.7 g/L was obtained. The glucose content values are in agreement with those previously obtained for pure hydrolysates of potato waste [16] and are higher than those reported in a previous study of acid hydrolyzed potato peelings [18]. The glucose in the medium came mainly from the hydrolysis of potato starch and in a small proportion from bagasse cellulose [16,30]. The small number of other monosaccharides detected in the hydrolysates was due to the presence of pectic polysaccharides and the hemicellulosic polysaccharides of grape bagasse [30]. For this reason, these monosaccharides were not detected in the control culture medium (Figure 2).

Glucose consumption can be related to both bacterial growth and BC production since it is the main carbon source in the medium. Comparing the glucose consumption of the GP medium with the control medium, a sharp decrease in glucose content in the GP medium was observed on the 3rd day, and this decrease took place in the control on the 2nd day. This could be due to the fact that the bacteria needed more time to adapt themselves to the new medium, but once the bacteria adapted, the glucose consumption was higher compared to the control. Thus, in the GP medium, all the glucose was consumed in less than 8 days, and in the control, it was on day 10. The presence of micronutrients in the GP medium led to an increase in the activity of the bacteria, with an increased rate of consumption of the main carbon source (glucose).

BC production in the GP medium was much higher than BC production in the control medium (Table 1 and Table 2). After 6 days, a BC concentration of 0.78 g/L was reached in the control medium, obtaining an average of 0.02 g of dry BC per 25 mL of medium. However, this production was five times higher in the GP medium, reaching a BC concentration of 4.0 g/L and an average of 0.10 g of dry BC per 25 mL of GP medium. Note that, in the control medium on day 2, unlike the GP medium, BC production slowed down without increasing significantly during the next 8 days (Table 1 and Table 2). The BC concentration values achieved in the present study were higher than those observed in previous work using grape pomace extract or potato residues. Grape pomace as a carbon source and corn steep liquor as the main nitrogen source needs 18 days of incubation to reach a concentration of over 4 g/L [10]. In the case of HS medium with white grape bagasse replacing the carbon source, incubated for 14 days at 28 °C, the BC concentration was 1.2 g/L [12]. In culture media containing pure xylose, glucose: galactose (1:1), lactose or glycerol, the BC production was 0.26, 2.60, 0.31 and 2.07 g/L, respectively [11]. The application of acid hydrolysates of potato peel waste for BC fermentation resulted in BC production of 1.21–2.61 g/L after 96 h of incubation [18].

Additionally, the higher activity of the bacteria in the GP medium and the increased consumption of glucose promoted higher production of gluconic acid (Table 1 and Table 2, and Figure 1 and Figure 2). Glucose dehydrogenase located in the cytoplasmic membrane of *K. xylinus* oxidizes glucose to gluconic acid, resulting in decreased conversion of glucose to BC [21]. In addition, gluconic acid promotes a significant decrease in the pH of the culture medium, which may hinder BC synthesis [21,34,35].

The times of greatest glucose decrease corresponded to the times of highest gluconic acid concentration in the medium, being 24.7 g/L in the GP medium and 15.4 g/L in the control medium. Note that, despite starting from similar values of glucose concentrations, the production of gluconic acid in the GP medium was 1.6 times higher than that in the control medium. A higher production of gluconic acid may be related to higher growth and activity of *K. xylinus* bacteria in the GP medium than those in the control medium. The presence of micronutrients and antioxidant components in bagasse are able to enhance bacterial growth by increasing glucose consumption and BC and gluconic acid production. The obtained results are in agreement with those observed in previous studies in which the presence of antioxidant components in the culture medium, such as vitamin C or lignosulfonate, improved BC production yields [33,36].

The synthesis of gluconic acid leads to a decrease in the pH of the culture medium that could hinder BC biosynthesis [21], as can be seen in the pH values of the media shown in Table 1 and Table 2.

The pH of the media showed a sharp decrease on day 3 (medium control) and day 4 (medium GP) and a decrease of almost 50% of the initial glucose level that coincided with the maximum peaks of gluconic acid concentrations in both media. After 4 days of incubation time, the concentration of gluconic acid began to steadily decrease from 24.7 g/L to 9.7 g/L in the GP medium and from 15.4 g/L to 3.6 g/L in the control medium on day 10. In addition, the pH increased slightly from 3.5 to 3.8 in the GP medium and from 3.2 to 3.4 in the control medium as gluconic acid was consumed. The greatest increase in cellulose production occurred in the first few days (Table 1 and Table 2), overlapping with the highest glucose consumption; thereafter, the increase was slower until reaching a maximum concentration of BC. This steady increase in BC concentration, complemented by a decrease in gluconic acid, indicated that the gluconic acid by-product could be used by *K. xylinus* to produce BC, as reported elsewhere [21,35].

The cellobiose content of both media increased slightly from 3.3 to 13.3 g/L and from 0.4 to 2.9 g/L as the incubation period advanced, being higher in the GP medium. Cellobiose is the structural unit of cellulose molecules and is also a product in the hydrolysis of cellulose [37]. This increase in cellobiose concentration could be related to the increase in the BC content in the plates, being higher in the samples with the GP medium. In the case of the monosaccharides, xylose and arabinose, they were only observed in the GP medium, as they were components derived from the hydrolysis of pectin and hemicelluloses. No variations in xylose concentration were observed throughout the incubation time. However, arabinose was consumed by day 6, when glucose concentrations reached minimum values (2.5 g/L) before being completely consumed. Thus, *K. xylinus* began to consume arabinose when glucose was close to being completely consumed.

The main drawback of acid hydrolysis is the generation of degradation products, such as furfural or 5-(hydroxymethyl)-2-furaldehyde (HMF). These by-products are microbial growth inhibitors and must be controlled under lethal concentrations to allow posterior fermentation. HMF is generated as a degradation product from glucose. Furfural is generated as a degradation product from pentose (xylose and arabinose) and can be found, for example, in grape seeds or in hemicellulosic portions [16,23,38]. The acid hydrolysis of the bagasse–potato mixture resulted in the appearance of HMF (241.6 mg/L) and furfural (7.9 mg/L). Note that, in both cases, as *K. xylinus* fermentation progressed, the concentration of both compounds decreased, indicating that the *K. xylinus* bacteria were able to consume HMF and furfural at the concentration level of the case study. HMF reached minimum values (0.7–1.3 mg/L) on day 6 of fermentation, and furfural was completely consumed by day 3. A similar behavior was observed in the control, in which only HMF (1.8 mg/L) was detected. Furfural was not detected due to the absence of hemicellulosic components in the medium. A significant inhibitory effect of HMF and furfural on the activity of *K. xylinus* was reported at a concentration of 2 g/L of HMF and 0.4 g/L of furfural [39]. In the GP medium, the concentration of both components was much lower, not having to affect BC production performance. Thus, no detoxification process of the hydrolysate was necessary before further use. However, no evidence of the *K. xylinus* consumption of HMF and furfural at low concentrations was found in the literature.

Regarding the drained BC weight, the pellicles obtained from the GP medium, the drained weight of the samples increased from 0.48 to 5.28 g as the incubation time increased, in accordance with the increase in the BC concentration. However, in the control medium, on day 2, the drained weight remained between 2.09 and 2.71 g during the whole fermentation time, in agreement with the observed data of dry weight and BC concentration, due to the interruption in BC production.

The swelling capacity data (Table 1 and Table 2) indicate that, as the BC concentration increased, the moisture decreased. Thus, for dry pellicle values between 0.009 and 0.020 g, the swelling capacity remained between 13,352 and 28,169%. These values were reduced by more than eight times with increasing BC concentrations, obtaining a value of 3463% for 0.09 g dry samples. Likely, as the BC concentration increased, the sizes of the voids free to hold water decreased.

Overall, 6 days was enough to reach the maximum values of BC synthesis, because, after 6 days, the increase in BC is not relevant. At this period, in the GP medium, the estimated BC productivity was 0.028 g/L·h, the efficacy was 0.12 g/g and the yield was 0.13 g/g. However, in the control, the values were considerably lower, obtaining a productivity of 0.005 g/L, an efficacy of 0.03 g/g and a yield of 0.03 g/g. The significant difference in the parameters assessing BC synthesis showed enhanced production of BC in the medium from Garnacha bagasse and potato waste compared with the synthetic medium (control). The results indicate that the nutrients that were present in the proposed natural medium enhanced *K. xylinus* growth and BC production.

### 3.2. Characterization of Bacterial Cellulose Films

**Figure 3:**
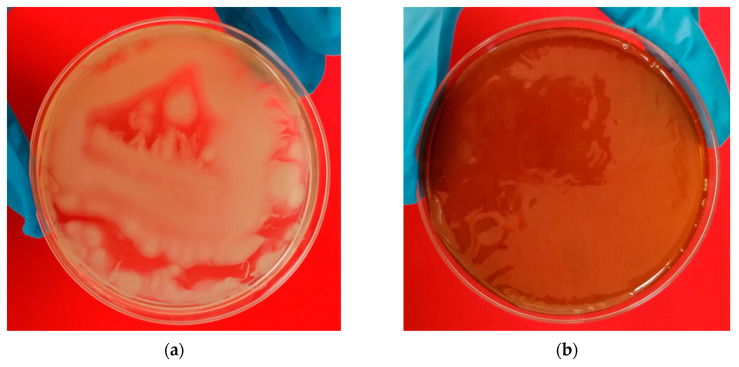
Images of bacterial cellulose films formed at the air–liquid interface of the culture medium: (**a**) Bacterial cellulose film growing in control culture medium showing non-homogeneous growth on the free surface of the Petri dish; (**b**) Bacterial cellulose film synthesized in bagasse–potato culture medium growing homogeneously covering the Petri dish surface.

The physicochemical properties of BC pellicles obtained from the culture medium from the studied agro-food waste were evaluated. Unlike the films from the natural GP culture medium, the films synthesized in the control medium did not homogeneously form a film along the surface of the plate, as shown in Figure 3a. The films synthesized in the control culture medium were irregular and brittle, and it was not possible to dry them and obtain samples with homogeneous thickness for their characterization.

Several Petri dishes with a diameter of 210 mm were prepared with GP culture medium and were incubated for 6 days at 30 °C under static conditions (Figure 3b). The BC membrane formed at the air–liquid interface was removed and was subsequently purified, washed and dried. Once the samples had been conditioned for at least 5 days at the specific relative humidity conditions for each test, they were measured.

The outstanding antioxidant capacity values of the films obtained confirmed the presence of the components of the GP culture medium in the polymer matrix despite the purification and washing processes to which the samples were subjected. The antioxidant capacity of film extracts obtained with ethanol and methanol was analyzed, observing TPC values of 0.31 and 1.32 mg GAE/g dried film, %DPPH^•^ of 57.24 and 78.00% and %ABTS^•+^ of 89.49 and 86.94%, respectively. The components with antioxidant capacity came mainly from bagasse hydrolysate with antioxidant capacity, as indicated above.

The obtained results for mechanical properties (tensile and puncture), water vapor permeability, moisture content at standard conditions of 57% RH and swelling capacity are presented in Table 3.

Comparing the results shown in Table 3 with previously published results for BC films with a similar thickness (0.02 mm) obtained from *K. xylinus* from a commercial medium with 10% glucose and 1% yeast extract incubated for 8 days at 30 °C [26,28], slight differences were observed. The BC films obtained from the synthetic medium showed a TS value of 20.76 MPa, %E of 2.28% and a Young’s modulus of 1043.88 MPa [28]. Considering the different incubation conditions, the films obtained from the GP medium showed higher breaking strength but lower deformation and elongation capacity. Likely, despite the purification process, some components of the bagasse–potato culture medium, such as phenolic compounds and antioxidants, remained embedded in the polymeric matrix of the BC, leading to a cross-linking effect. This effect could be seen most clearly in the puncture properties. Films from the GP medium showed higher BS and DB values (159.31 g and 0.70 mm) than those of films obtained from the synthetic medium (58.88 g and 0.39 mm) [26] despite having a lower thickness.

On the other hand, the obtained films showed lower permeability values of 3.40 × 10^−12^ g/m·s·Pa compared with films previously obtained from the synthetic medium (2.38 × 10^−11^ g/m·s·Pa) [28]. The assembly pattern of BC fibers could be altered depending on the bagasse–potato components suspended in the medium, obtaining a modified BC pellicle at a morphological level. The compound in the medium could modify the conformation of the BC chains and lead to new physic interactions between the supplement and BC. The supplementation effect on the structural film properties could extend to the rest of the functional properties of the film, such as mechanical resistance and the permeability of the films [40]. In the present study, cellulose synthesis in the GP medium could result in a more closed and compact polymeric structure, which hindered the passage of water molecules through the matrix, resulting in lower WVP values. Moreover, these permeability values are lower than those shown by other polysaccharide matrices, extending the applicability of this BC matrix [41].

The moisture content of the samples obtained under standard conditions (10.07%) was higher than that previously observed in films obtained in synthetic media (1.82%) [28]. This difference could be mainly due to the presence of remains with hydrophilic properties in the culture medium. These hydrophilic remnants in the BC matrix led to a higher swelling capacity of the BC matrix for the GP medium compared with the swelling capacity of a pure BC film (364.78%) with a thickness of 0.002 mm [26]. Additionally, the swelling capacity of the dried films (1053.27%) was lower than that observed in BC films removed directly from the Petri dishes (Table 1 and Table 2). This is because, once BC films are dried, the structure closes and the physical interactions between the polymeric chains increase, not being able to reach swelling capacity values similar to those of a non-dried film [42].

The UV barrier properties of the BC samples from the GP culture medium were calculated from the transmittance values in the UV region. The transparency, opacity and color of the pellicles were determined from the transmittance values in the visible region. The samples showed a significant barrier capacity against UV radiation, giving mean values of percent transmittance ranging from 0.02 to 0.34%. The UV blocking capacity of the BC films were enhanced by structural modifications or components in the GP medium that remained between the polymer matrix. In previous work, BC films from synthetic media showed UV barrier values ranging from 1.35 to 5.61% [4]. These remains of the GP medium trapped between the cellulose chains also modified the opacity and color properties of the films. The transparency of the film (26.97 ± 51.47) remained at values similar to those previously observed in synthetic BC medium films [4], but the opacity (214.57 ± 61.95) increased more than 10-fold. In addition, the lightness of the samples (L* value of 14.16 ± 11.91) decreased, and the redness (a* value of 4.84 ± 1.63) and yellowness (b* value of 11.05 ± 6.21) increased.

## 4. Conclusions

This work demonstrates that it is possible to improve the production and yield of BC from a natural medium based on bagasse and non-commercial potato waste. The proposed low-cost culture medium could be an alternative to give added value to the by-products of the agri-food industry and to obtain a raw material with potential use in the food, cosmetic, medical and pharmaceutical industries. Nutrients of the culture medium, due to the presence of bagasse, increased BC production yield compared with the control synthetic medium. The enriched medium also promoted increased activity of the bacteria, converting glucose to gluconic acid. The production of gluconic acid reduced the availability of glucose and lowered the pH of the medium, hindering BC synthesis. The ability of *K. xylinus* to consume HMF and F at low concentrations was observed, which raises interesting applications that deserve to be deeply studied in future work.

The obtained films showed permeability values in orders of one and two magnitudes lower than the permeability properties of most of the other polysaccharide-based films. In addition, components with antioxidant capacity from Garnacha bagasse were retained between the BC chains during polymer synthesis, providing antioxidant capacity and functionality to the films.

The UV radiation blocking capacity of the samples, together with their antioxidant capacity, edibility and water insolubility, make BC films from GP media a material with potential applications in direct food contact to retard oxidation reactions that may modify the organoleptic properties of the product.

## Figures and Tables

**Table 1:** Parameters analyzed at each incubation time in bagasse–potato culture medium: pH, bacterial cellulose (BC) drained weight (g), BC dried weight (g), BC concentration (g/L), BC swelling capacity (%S) and gluconic acid concentration (g/L).

Time	pH	Drained Weight	Dried Weight	BC	BC %S	Gluconic Acid
Day		g	g	g/L	%	g/L
0	5.20 ± 0.01 ^a^	-	-	-	-	6.49 ± 0.19 ^a^
2	4.68 ± 0.11 ^b^	0.48 ± 0.15 ^a^	0.01 ± 1.18 × 10^−3 a^	0.23 ± 0.05 ^a^	8611 ± 3001 ^abe^	9.43 ± 0.74 ^b^
3	3.57 ± 0.04 ^cd^	2.15 ± 0.10 ^b^	0.02 ± 2.73 × 10^−3 a^	0.67 ± 0.11 ^a^	12,935 ± 2551 ^a^	24.42 ± 1.47 ^c^
4	3.46 ± 0.01 ^c^	3.02 ± 0.14 ^bc^	0.041 ± 4.01 × 10^−3 a^	1.64 ± 0.16 ^ae^	7318 ± 745 ^bc^	24.72 ± 0.33 ^c^
5	3.50 ± 0.01 ^cd^	3.23 ± 0.50 ^bc^	0.09 ± 0.02 ^b^	3.66 ± 0.75 ^b^	3463 ± 419 ^c^	22.39 ± 0.43 ^c^
6	3.55 ± 0.01 ^cde^	3.71 ± 0.19 ^ce^	0.10 ± 0.01 ^b^	4.00 ± 0.49 ^b^	3640 ± 393 ^c^	19.47 ± 1.22 ^d^
7	3.58 ± 0.01 ^bde^	4.69 ± 0.56 ^ef^	0.08 ± 0.01 ^b^	3.10 ± 0.33 ^bce^	5952 ± 217 ^ec^	15.79 ± 0.96 ^e^
8	3.64 ± 0.05 ^bef^	4.80 ± 0.41 ^ef^	0.11 ± 0.03 ^b^	4.32 ± 1.07 ^bc^	4615 ± 1646 ^ec^	13.36 ± 1.19 ^f^
9	3.70 ± 0.05 ^bf^	5.18 ± 0.20 ^f^	0.10 ± 0.01 ^b^	4.14 ± 0.48 ^b^	4963 ± 722 ^ec^	12.00 ± 0.82 ^fg^
10	3.80 ± 0.05	5.28 ± 0.76 ^f^	0.09 ± 0.01 ^b^	3.71 ± 0.30 ^b^	5658 ± 1276 ^ec^	9.86 ± 1.32 ^g^

Values are expressed as mean ± standard deviation (SD). Different letters in the same column indicate significant differences (*p* > 0.05).

**Table 2:** Parameters analyzed at each incubation time in control culture medium: pH, bacterial cellulose (BC) drained weight (g), BC dried weight (g), BC concentration (g/L), BC swelling capacity (%S) and gluconic acid concentration (g/L).

Time	pH	Drained Weight	Dried Weight	BC Concentration	BC %S	Gluconic Acid
Day		g	g	g/L	%	g/L
0	5.26 ± 0.01 ^a^	-	-	-	-	0.98 ± 0.15 ^a^
2	3.25 ± 0.03 ^bd^	2.47 ± 0.48 ^a^	0.009 ± 0.001	0.35 ± 0.02 ^a^	28,169 ± 4038 ^a^	11.95 ± 0.28 ^b^
3	3.17 ± 0.01 ^c^	2.71 ± 0.20 ^a^	0.010 ± 3.06 × 10^−4^	0.41 ± 0.01 ^a^	26,592 ± 1306 ^a^	15.40 ± 0.33 ^c^
4	3.17 ± 0.01 ^c^	2.61 ± 0.40 ^a^	0.010 ± 0.002	0.41 ± 0.07 ^a^	25,471 ± 1249 ^a^	14.53 ± 0.20 ^c^
5	3.15 ± 0.02 ^c^	2.29 ± 0.50 ^a^	0.011 ± 0.001	0.43 ± 0.05 ^a^	20,948 ± 4246 ^abc^	12.72 ± 0.31 ^b^
6	3.18 ± 0.02 ^bc^	2.61 ± 0.21 ^b^	0.020 ± 0.003	0.78 ± 0.13 ^b^	13,352 ± 1487 ^bc^	10.08 ± 0.93 ^d^
7	3.27 ± 0.01 ^d^	2.71 ± 0.55 ^a^	0.012 ± 0.004	047 ± 0.14 ^a^	22,496 ± 2653 ^abc^	7.88 ± 0.67 ^e^
8	3.27 ± 0.03 ^d^	2.65 ± 0.04 ^ab^	0.014 ± 0.004	0.54 ± 0.17 ^a^	20,787 ± 5874 ^ac^	6.11 ± 0.80 ^f^
9	3.35 ± 0.06 ^e^	2.39 ± 0.87 ^a^	0.011 ± 0.0031	0.44 ± 0.10 ^a^	21,205 ± 2961 ^a^	4.57 ± 0.36 ^g^
10	3.42 ± 0.02 ^f^	2.09 ± 0.44 ^ab^	0.013 ± 0.001	0.52 ± 0.02 ^a^	16,100 ± 3956 ^c^	3.61 ± 0.14 ^h^

Values are expressed as mean ± standard deviation (SD). Different letters in the same column indicate significant differences (*p* > 0.05).

**Table 3:** Physicochemical properties of bacterial cellulose films synthesized in bagasse–potato culture media.

Property	Value	Unit
Thickness	0.010 ± 0.001	mm
Tensile strength	22.77 ± 2.54	MPa
Percentage of elongation	1.65 ± 0.43	%
Young’s Modulus	910.46 ± 48.66	MPa
Burst strength	159.31 ± 47.99	g
Distance to burst	0.70 ± 0.14	mm
Water vapor permeability	3.40 × 10^−12^ ± 1.35 × 10^−13^	g/m·s·Pa
%W *	10.07 ± 1.55	%
Swelling capacity	1053.27 ± 45.59	%

* Moisture content at equilibrium (57% RH).

## Data Availability

The data presented in this study are available on request from the corresponding author.
